# Histomorphometry of Bone after Intentionally Exposed Non-Resorbable d-PTFE Membrane or Guided Bone Regeneration for the Treatment of Post-Extractive Alveolar Bone Defects with Implant-Supported Restorations: A Pilot Randomized Controlled Trial

**DOI:** 10.3390/ma15175838

**Published:** 2022-08-24

**Authors:** Roberto Luongo, Marco Tallarico, Elena Canciani, Daniele Graziano, Claudia Dellavia, Marco Gargari, Francesco Mattia Ceruso, Dario Melodia, Luigi Canullo

**Affiliations:** 1 Arthur Ashman Department of Periodontology and Implant Dentistry, NYU College of Dentistry, New York, NY 10010, USA; 2 Independent Researcher, 70100 Bari, Italy; 3 Department of Medicine, Surgery, and Pharmacy, University of Sassari, 07100 Sassari, Italy; 4 Department of Biomedical, Surgical and Dental Sciences, Università degli Studi di Milano, 20100 Milan, Italy; 5 San Pietro Hospital, 00189 Rome, Italy; 6 School of Dentistry, University of Sassari, 07100 Sassari, Italy; 7 Department of Periodontics and Implantology, University of Bern, 3000 Bern, Switzerland

**Keywords:** non-resorbable membrane, guided bone regeneration, d-PTFE, xenograft, dental implants, histomorphometry

## Abstract

**Aim**: The aim of the present study was to investigate quantitative histological examination of bone reconstructed with non-resorbable high-density polytetrafluoroethylene membrane (d-PTFE), left intentionally exposed in post extraction sockets grafted with anorganic bone material, and removed after four weeks, versus extraction and guided bone regeneration (GBR), performed two months later. **Materials and Methods**: This study was designed as a multicenter randomized controlled trial of parallel-group design. Patients were selected and consecutively treated in three centers in Italy. Patients randomly received intentionally exposed non-resorbable d-PTFE membrane (group A), or guided bone regeneration (group B), to treat post-extractive alveolar bone defects with implant-supported restorations. Outcomes were: the implant failure, any mechanical and biological complications, patient satisfaction, and qualitative and histomorphometric evaluation of the collected bone samples. **Results**: Eighteen patients were consecutively enrolled in the trial. Of these, six out of 18 patients were male. All the included patients were treated according to the allocated interventions, and no drop out occurred. No implant failure and no complications were experienced, and all the patients were fully satisfied with the function and aesthetic of their implant-supported restoration, without difference between groups. Morphological analysis revealed no sign of tissue reaction, such as fibrosis or necrosis. Regenerated bone was well mineralized in both groups, but it seemed more mature in group B than in group A. Three samples showed a minimal number of lymphocytes. Several blood vessels of small size occupied the medullary spaces, where the tissue resulted in more maturity, indicating the activity of the tissue in progress. The histomorphometric evaluation showed no statistically significant differences in the tissue volume fractions between the two groups of patients. **Conclusions**: With the limitation of the present study, buccal plate reconstruction with an intentionally exposed non-resorbable membrane is an effective and easy procedure for regenerating a resorbed buccal bone plate, reducing the need for guided bone regeneration.

## 1. Introduction

Loss of alveolar bone may be attributed to a variety of factors, such as endodontic pathology, periodontitis, facial trauma, and aggressive maneuvers during extractions [1,2]. Moreover, after tooth extraction, a cascade of biological events occurs, typically resulting in significant local anatomic changes, including reduced height and reduced width of the residual ridge [3,4]. Horizontal bone loss is generally the greatest and occurs more frequently on the buccal side [3]. Favorable implant success rates and peri-implant tissue responses may be achieved by placing implants immediately after tooth extraction, however, the continuing recession of the facial gingival tissue was observed [5]. In order to improve the aesthetic predictability of post-extractive implants, several studies and systematic reviews have been conducted to evaluate the efficacy of different socket-filling approaches involving different grafting materials, with or without barrier membranes [6,7,8,9,10,11,12,13,14,15,16]. In these studies, a plethora of biomaterials have been used, including autologous bone, bone substitutes (allografts, xenografts, and alloplasts), and autologous blood-derived products, and bioactive agents. However, recent clinical trials [11,12] and systematic reviews [13,14,15,16] failed to find evidence that a biomaterial and barrier membrane was superior over the others. With the limitations of this study, the use of high-density polytetrafluoroethylene non-resorbable (d-PTFE) membranes was safe and predictable, particularly in terms of keratinized tissue width [13].

The same studies encourage well-conducted randomized controlled trials.

Elian et al. proposed a simplified socket classification and a noninvasive approach to the grafting and management of sockets when soft tissue is present, but the buccal plate is compromised (Type II) [17]. Authors used collagen membrane and graft materials for the treatment of type II sockets. A recent systematic review concluded that PTFE membranes protect the grafting material and/or the initial healing clot from bacterial contamination, leading to successful regeneration without a significant risk of infection [14].

The aim of the present randomized controlled trial was to investigate the quantitative histological examination of bone reconstructed with d-PTFE membrane, left intentionally exposed in post extraction sockets grafted with anorganic bone material, and removed after four weeks, versus extraction and guided bone regeneration (GBR), performed two months later. The null hypothesis was that there were no statistical differences regarding the qualitative and histomorphometric evaluation of samples between groups. The null hypothesis was tested against the alternative hypothesis of difference. The present research was written according to the CONSORT guidelines.

## 2. Materials and Methods

This study was designed as a pilot, multicenter randomized controlled trial of parallel-group design, aimed to evaluate histomorphometry of bone and clinical parameters of patients with mandibular and maxillary hopeless teeth located between premolars, and require a delayed implant-supported single crown restoration. Patients were selected and consecutively treated in three centers in Italy from November 2018 to September 2020.

Three clinicians performed both surgical and prosthetic procedures. Calibration of the surgeons ensured they performed two adjunctive clinical cases each, in order to estimate and reduce potential surgical risks. This study was conducted in accordance with the principles outlined in the Helsinki Declaration of 1964 for biomedical research involving human subjects, as amended in the 64th WMA General Assembly, Fortaleza, Brazil, October 2013, and received ethical approval from “Ethical Committee Lazio 1”. Protocol number 23/CE Lazio 1, of 7 January 2020. Patients were duly informed about the nature of the study. A written informed consent form for surgical and prosthetic procedures, as well as for the use of the clinical and radiological data, were obtained for each patient before treatments started.

### 2.1. Inclusion and Exclusion Criteria

Any subject requiring at least one implant-supported single restoration between premolars, to replace a failed tooth with a damaged buccal bone plate but maintained soft tissue architecture (Type II according to Elian et al. [17]), being at least 18 years old, and able to sign an informed consent form, was considered eligible for this study and consecutively enrolled. Hopeless teeth were judged as follow: furcation involvement > II; mobility > II; PPD > 6 mm with the percentage of alveolar bone loss/root length ≥70%; persistent radiographic pathology and/or symptoms (e.g., pain, fistula, abscess) of endodontic origin and an uncertain prognosis; restorability.

Each patient contributed only with one procedure. The selected site had to have adjacent teeth/implants. Exclusion criteria were:

- General contraindications to oral surgery (such as ASA III and IV);
- Heavy smokers (≥ 10 cigarettes/day);
- Addiction to alcohol or drugs;
- Acute and chronic infections in the site intended for implant placement;
- Poor oral hygiene (full mouth bleeding and full mouth plaque index higher than 25%);
- Pregnancy or nursing;
- Psychiatric therapy;
- Patients treated or under treatment with intravenous amino bisphosphonates;
- Previous radiotherapy of the oral and maxillofacial region within the last 5 years;
- Patients unable to commit to the scheduled follow-up.

### 2.2. Clinical Procedures

Potentially eligible patients were evaluated clinically, and their medical histories were recorded. During the visit, preoperative periapical radiographs, study models, and pictures were obtained, and periodontal screening was recorded.

Patients underwent professional oral hygiene prior to the surgery and received prophylactic antiseptic (0.2% chlorhexidine mouthwash one minute prior to the surgery) and antibiotic therapy (two gr of amoxicillin and clavulanic acid, or clindamycin 600 mg if allergic to penicillin, one hour prior to surgery). All patients were treated under local anesthesia using articaine hydrochloride with adrenaline 1:100,000 (Orabloc, Pierrel, Milan, Italy). Teeth extractions were performed flapless, as atraumatically as possible. Multiple-rooted teeth were sectioned at the furcation, and the roots were individually extracted. Afterward, the residual extraction socket was washed with saline solution, and it was debrided thoroughly from granulation tissue and residual periodontal ligament fibers. Finally, the bony wall was evaluated with the aid of a periodontal probe (PCPUNC156, Hu-Friedy). Afterward, patients who fulfilled all the inclusion criteria were definitively enrolled, and a sequentially numbered, sealed envelope corresponding to the patient recruitment number was opened by a blinded independent assistant to get to know the allocation group.

**Figure 1:**
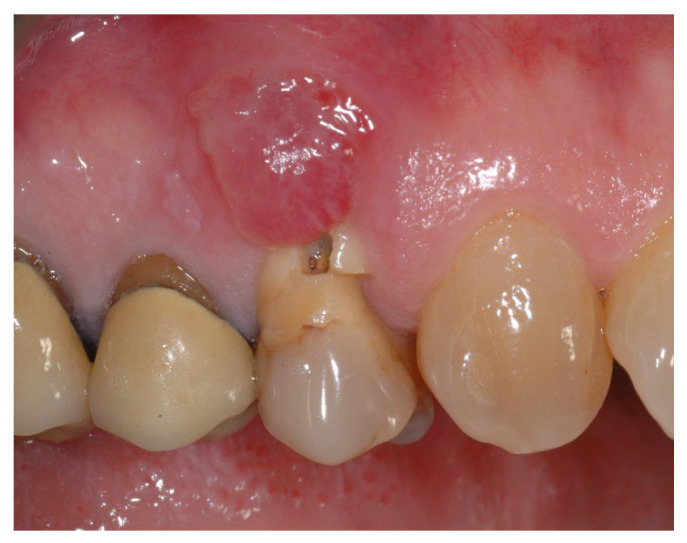
Group A, initial scenario.

**Figure 2:**
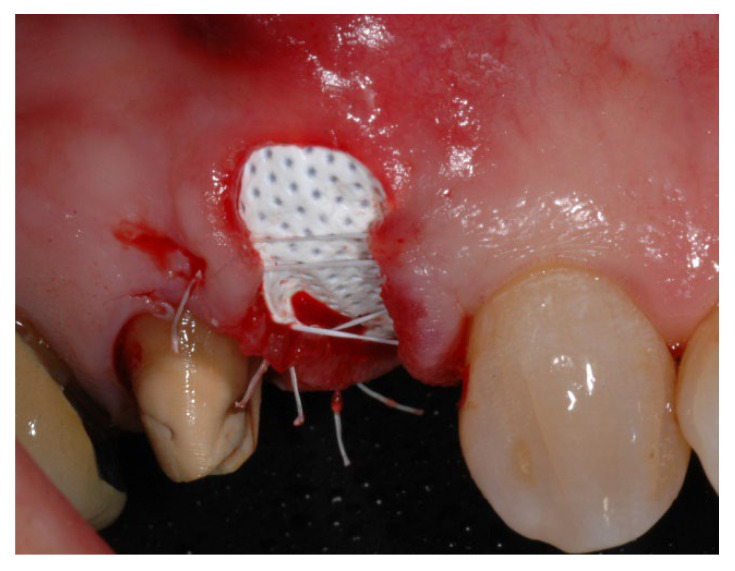
Group A, non-resorbable d-PTFE membrane left intentionally exposed after tooth extraction and socket preservation.

**Figure 3:**
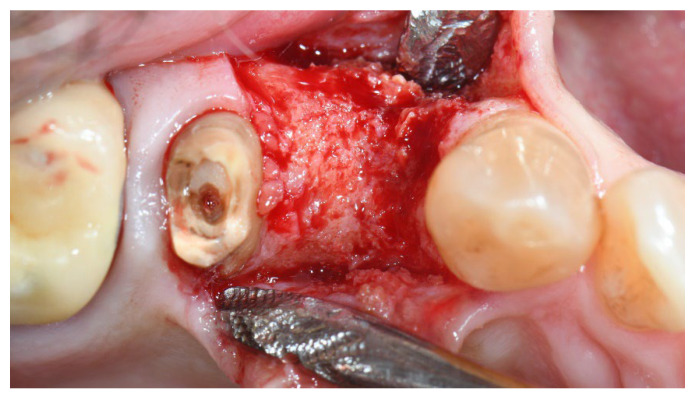
Group A, bone healing at implant placement.

**Figure 4:**
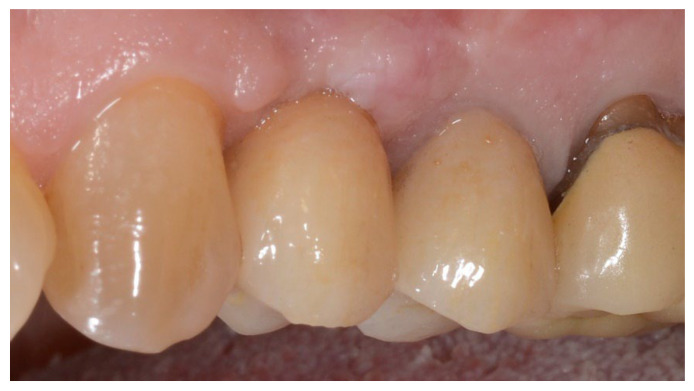
Group A, definitive prosthesis delivery.

In group A, socket preservation with intentionally exposed non-resorbable d-PTFE membrane (test group, Figure 1, Figure 2, Figure 3 and Figure 4), the residual alveolar socket was grafted with porcine-derived cancellous anorganic bone material (0.25–1 mm particles, Zcore, DeOre s.r.l., Negrar [Vr], Italy). Then, a non-resorbable, dense-polytetrafluoroethylene (d-PTFE) membrane (Cytoplast TXT1224, DeOre) was shaped according to the dimension of the residual socket, and it was inserted into a buccal and lingual pocket. Finally, a horizontal mattress suture (Cytoplast PTFE Suture 4-0, DeOre) was placed to secure the membrane and stabilize it to the soft tissue margins. The application of the non-resorbable d-PTFE membrane did not require primary closure via buccal flap advancement. The sutures were removed between 10 and 14 days post-surgery, and the non-resorbable d-PTFE membrane was removed between the fourth and the fifth weeks after surgery. After that, the wound was left to heal for about five months, allowing by the process of re-epithelialization.

**Figure 5:**
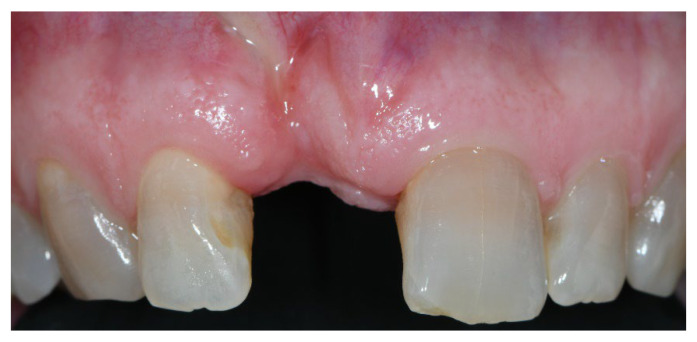
Group B, tissues healing eight weeks after tooth extraction.

**Figure 6:**
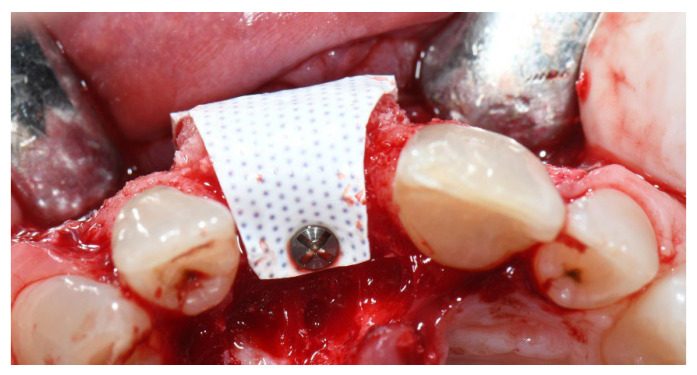
Group B, guided bone regeneration.

**Figure 7:**
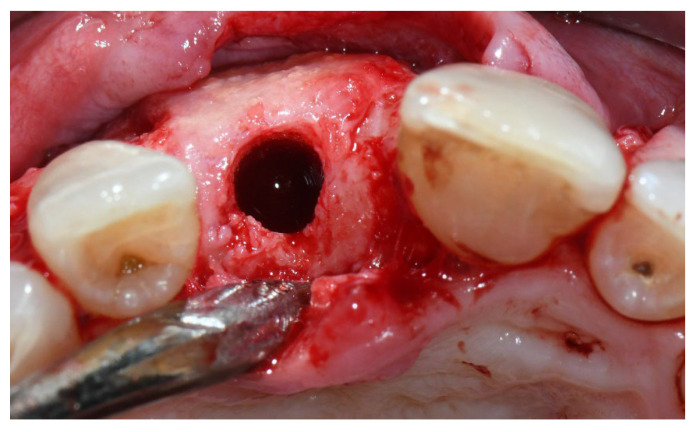
Group B, implant site preparation.

**Figure 8:**
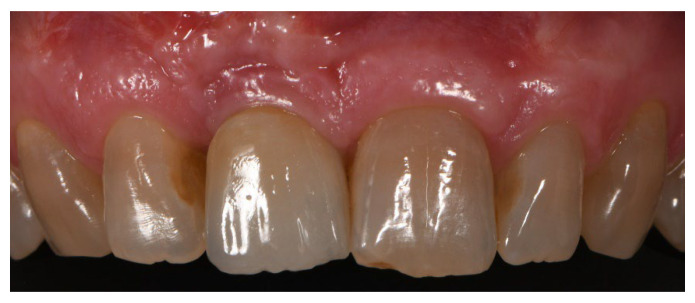
Group B, definitive prosthesis delivery.

In group B, GBR (control group, Figure 5, Figure 6, Figure 7 and Figure 8), just a fibrine sponge was inserted into the socket to stabilize the blood clot.

In both groups, 1g of amoxicillin (or 300 mg of clindamycin) was administered every 12 h for six days after tooth extraction and bone reconstruction [18]. Paracetamol 500 mg plus codeine 30 mg were prescribed as needed. Patients were instructed not to take them in the absence of pain. Chlorhexidine spray 0.2% twice a day for two and five weeks was prescribed in groups A and B, respectively. A soft diet was recommended for two weeks after surgical procedures in both groups.

In group B, eight weeks after tooth extraction, an intrasulcular incision was made using a no. 15C Bard-Parker blade, and a full-thickness flap was elevated beyond the mucogingival junction and at least 5 mm beyond the bone defect. Two vertical incisions were placed at least one tooth away from the area to be augmented. Multiple decortication holes at the recipient site were performed with a round bur. The residual alveolar socket was grafted with particles of porcine-derived cancellous anorganic bone material (Zcore, DeOre). Then, a collagen membrane (Cytoplast RTM Collagen, DeOre) was shaped according to the regenerated bone defect and was fixed with three to five titanium pins (Supertack, MCbio s.r.l., Lomazzo, Italy), on the buccal and lingual/palatal sides. The membrane was trimmed to the entire graft volume, one to two millimeters before the adjacent teeth surface. A periosteal incision was performed between the two vertical incisions to allow a completely tension-free closure of the flap. The flaps were then sutured in two layers in order to prevent exposure to the membrane (Cytoplast PTFE Suture 4-0). Horizontal mattress sutures were first placed 4 mm from the incision line; then, single interrupted sutures were placed to close the edges of the flap. Vertical incisions were sutured with single interrupted sutures. The single interrupted sutures were removed between 10 and 14 days post-surgery, and mattress sutures were removed two to three weeks after surgery. One g of amoxicillin (or 300 mg of clindamycin) was administered every 12 h for eight days after tooth extraction and bone reconstruction. Paracetamol 500 mg plus codeine 30 mg were prescribed as needed. Patients were instructed not to take them in the absence of pain. Chlorhexidine spray 0.2% twice a day for two and five weeks was prescribed in groups A and B, respectively. A soft diet was recommended for 2 weeks after surgical procedures in both groups.

In both groups, six to eight months after tooth extraction (group A and B, respectively), dental implants (Premium, Sweden and Martina, Due Carrare, PD, Italy) were placed. In none of the cases was it necessary to carry out an additional GBR. The amount of bone was judged sufficient to place an implant of 3.8 mm of diameter and 10 to 11.5 mm of length. Flap design was performed according to the clinical scenario and the patient’s requirements. Before implant site preparation, a calibrated trephine bur with a 3.0 mm external diameter was used to collect a core sample for histologic analysis. The implants were submerged for three months. Three months after implant placement, a screw-retained temporary restoration was delivered. Finally, two to three months after initial loading, a definitive, screw-retained, CAD/CAM, metal-free restoration was delivered. Occlusion was adjusted, and follow-up visits were scheduled every four months.

### 2.3. Outcome Measures

- Implant failure was defined as implant mobility and/or any infection dictating implant removal, implant fracture, or any other mechanical complication that renders the implant useless. In addition, the stability of each implant was measured manually by tightening the abutment screw at delivery of definitive crowns, or by assessing the stability of the implant-supported crown using the handle of two metallic instruments at each follow-up.
- Any mechanical and biological complications were recorded during the entire follow-up by period. The same calibrated operators, who performed all the surgical and prosthetic procedures, evaluated implant failures and complications.
- Patient satisfaction was evaluated by blinded operators in each center, not previously involved in the study. At delivery of definitive crowns, the independent outcome assessor asked the patient the following questions (possible answers: “yes” or “no”):
  ○ Are you satisfied with the function of your implant-supported tooth?
  ○ Are you satisfied with the aesthetic outcome of your implant-supported tooth?
  ○ Would you undergo the same therapy again?
- Quantitative and qualitative analyses were performed on anonymized samples by blinded pathologist. Qualitative and histomorphometric evaluation. After collection, the bone samples were fixed in 10% formalin. The samples were dehydrated with an increasing ethanol scale. After dehydration, the samples were infiltrated with a methacrylate resin and subsequently embedded in resin (Technovit 7200, Bio Optica, Milan). All the specimens were polymerized using a polymerization machine (Exakt 520, Exakt Norderstedt, Germany). The obtained blocks were cut by means of a sawing machine with a diamond blade (Micromet, Remet, Bologna, Italy). The sections were mounted on plastic slides using a gluing machine (Exakt 402, Exakt Norderstedt, Germany) and a gluing resin (Technovit 7210, Bio Optica, Milan, Italy) and then ground to a thickness of 100 μm using a grinding machine (LS2 Remet, Bologna, Italy). Two representative longitudinal sections for each sample were obtained, stained with Toluidine Blue and Pyronine Yellow to highlight the different phases of bone mineralization, and digitally acquired at the total magnification of 400× using a high-resolution scanner (Nanozoomer S60, Hamamatsu) [19].
- The qualitative assessment aimed to detect the amount of inflammatory, fibrous, and fatty tissue infiltrate and eventual areas of necrosis following the indication reported by ISO standard 10993. The histomorphometric evaluation of tissue volume fractions were performed using the stereologic method. A digital counting grid was used on each section to calculate the intersection points that fall down on each kind of tissue (lamellar bone, woven bone, osteoid matrix, biomaterial, and medullary spaces), and the volume fraction percentages were obtained by the ratio between the intersection points that fall down on each type of tissue and the total intersection points of the grid.

### 2.4. Statistical Analysis

No sample size was calculated. It was decided to recruit 30 patients, since this number was within the capability of the present research groups. Three randomization lists were created online at the Random Sequence Generator website (https://www.random.org). Only one of the investigators (FMC), not involved in the selection and treatment of the patients, was aware of the random sequence and could have access to the randomization lists stored in a password-protected portable computer. The random codes were enclosed in sequentially numbered, identical, opaque, sealed envelopes. Envelopes were opened sequentially after eligible patients signed the informed consent; therefore, treatment allocation was concealed to the investigators in charge of enrolling and treating the patients. Due to the nature of the study, after groups allocation, surgeons were not blinded. Nevertheless, samples were anonymized, and histology laboratory was blinded during the analyses.

All data analysis were carried out according to the pre-established analysis plan. Descriptive analysis was performed using the mean ± standard deviation (SD) using (Numbers for Mac V. 11.0 [7030.0.94], Apple Inc., Los Altos, California, USA). Dichotomous outcomes were compared between groups using. Comparisons between continuous variables were performed using an independent t-test. All statistical comparisons were conducted at the 0.05 level of significance. The patients were used as the statistical unit.

## 3. Results

**Figure 9:**
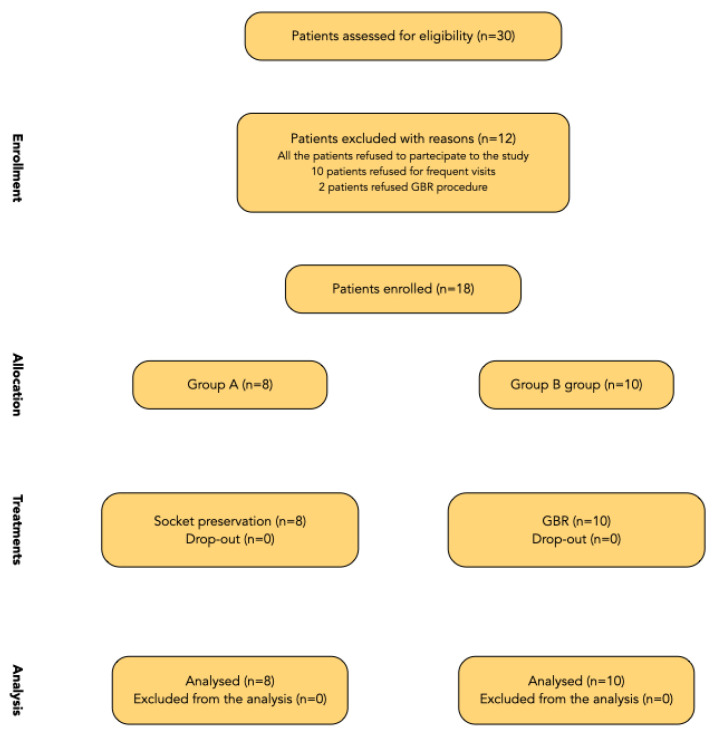
Flow chart of the treated patients.

A flow chart of the treated patient is reported in Figure 9. Patients had to be recruited and treated using similar procedures in three different centers, and each center was supposed to recruit and treat 10 patients. However, only one center recruited all the planned patients, while the other two centers recruited four patients each. Reasons for not including 12 patients were: frequent check-up visits (10 patients), and a refusal for guided bone regeneration (two patients). Finally, 18 patients were consecutively enrolled in the trial belonging to a cohort of 30 patients initially screened for eligibility. Eight patients were randomly allocated to group A, while 10 patients were randomly allocated to group B. Of these, six out of 18 patients were male.

All the included patients were treated according to the allocated interventions. The first patient was treated on November 2018, while the last surgical treatment was started in September 2020. No patient dropped out of the trial, and no deviations from the original protocol occurred; therefore, all the patients received the final crowns. The mean follow-up after prosthesis delivery was 6 to 30 months. The mean age of the patients was 56.9 ± 11.9, none of whom were smokers. Eighteen implants were placed, distributed by eight in the test group (socket preservation, group A) and 10 in the control group (GBR, group B). Data of all the included patients were evaluated in the statistical analyses. When comparing the tested groups, there is no unbalancing between them, including teeth position and distribution (Table 1).

### 3.1. Implant Failure, Complications, and Patients’ Satisfaction

No implant failure and no complications were experienced. In addition, all the patients were fully satisfied with the function and aesthetic of their implant-supported restoration, and no differences were experienced in their perception of the therapy, so all the patients would undergo the same therapy.

### 3.2. Histomorphometric Analysis

**Figure 10:**
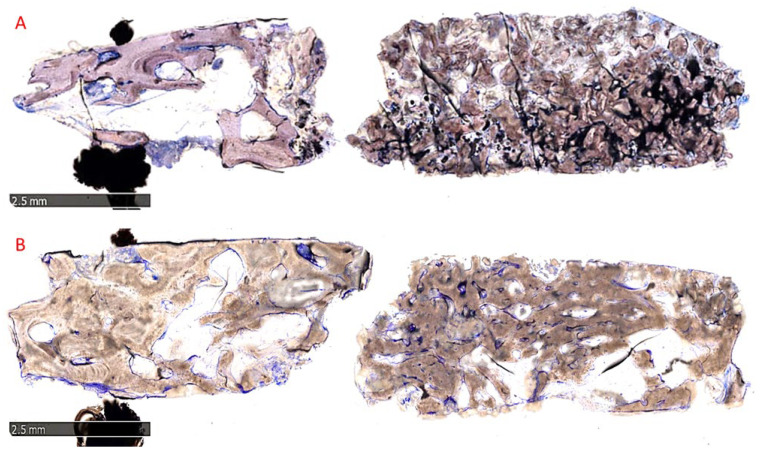
Overview of two representative samples: (**A**) Socket preservation; (**B**) GBR. A large amount of regenerated bone surrounds grafted blocks in the coronal portion of the biopsies (right side), while in the apical portion (left side), basal bone is observable in both groups. Total magnification 25×, Toluidine Blue and Pyronine Yellow staining.

Morphological analysis revealed no sign of tissue reaction, such as fibrosis or necrosis. Regenerated bone was well mineralized in both groups, but it seemed more mature in group B than in group A as observable in Figure 10A,B, which shows representative samples’ overviews.

**Figure 11:**
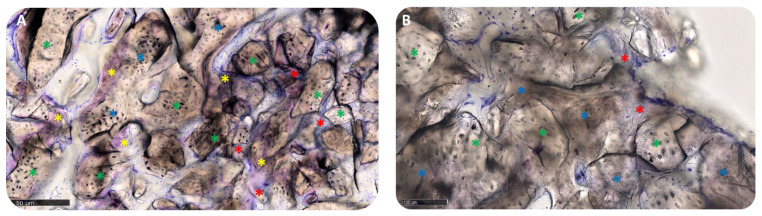
Osseointegration. (**A**) Socket preservation; (**B**) GBR. Biomaterial blocks (green asterisks) are surrounded by matrix at different stages of mineralization (blue asterisks: lamellar bone; yellow asterisks: woven bone; red asterisks: osteoid matrix). The staining shows in brown the bone matrix at a higher level of mineralization (particularly in photo B), while the bone matrix still in the phase of calcification is colored in purple. Total magnification (**A**,**B**): 200×. Toluidine Blue and Pyronine Yellow staining.

In group A, the grafted particles were wholly embedded in the bone matrix and appeared to be undergoing intense remodeling, with an intermediate degree of mineralization. No gaps were visible at the interface between residual blocks and newly formed bone (Figure 11A). In group B, the bone matrix that surrounded the biomaterial particles presented a high level of mineralization and osseointegration with a balance of bone formation (anabolic phase) and bone degradation (catabolic phase), confirmed by the presence of some fronts of bone remodeling and a few osteoclasts (Figure 11B).

**Figure 12:**
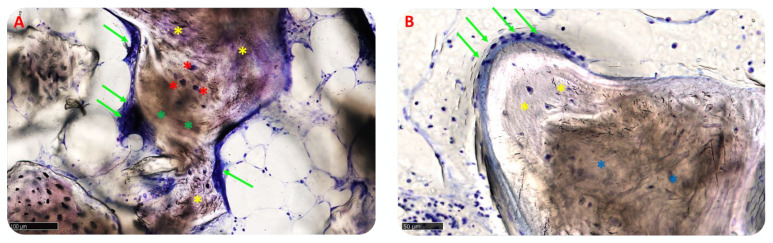
Bone cells. (**A**) Socket preservation. The osteoid matrix (in blue) is rich in osteoblasts (green arrows) that deposit the new bone matrix and then become osteocytes (red asterisks) remaining englobed into the newly formed matrix. Green asterisks represent biomaterial blocks. Total magnification: 200×; (**B**) GBR. Green arrows indicate osteoblasts in the osteoid matrix that surrounds woven bone (yellow asterisk) in close contact with newly lamellar bone (blue asterisks). Total magnification: 350×. Toluidine Blue and Pyronine Yellow staining.

The matrix was in the active anabolic phase: bone areas were made of newly formed lamellar, mineralized bone, with osteocytes into calcified matrix and front of remodeling populated by active osteoblasts and few osteoclasts, particularly in group A (Figure 12A,B).

**Figure 13:**
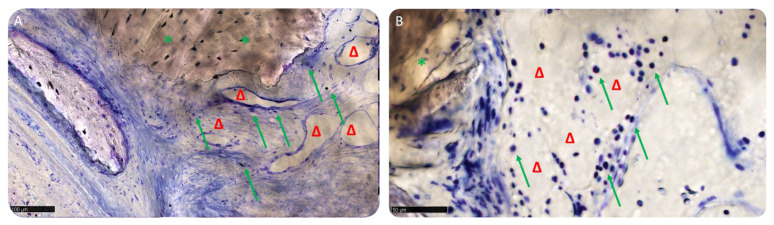
Inflammatory cells. (**A**) Socket preservation: green arrows indicate lymphocytic cells, green asterisks mark the biomaterial blocks. Δ indicates blood vessels. Total magnification: 200×; (**B**) GBR. Green arrows indicate lymphocytic cells. Green asterisks mark biomaterial blocks. Δ indicates blood vessels. Total magnification: 450×. Toluidine Blue and Pyronine Yellow staining.

Three samples showed a minimal lymphocyte, while the remaining samples of both groups had only some scattered inflammatory cells (Figure 13A,B). Several blood vessels of small size occupied the medullary spaces, where the tissue resulted in more maturity, potentially indicating activity of the tissue in progress.

The histomorphometric evaluation showed no statistically significant differences in the tissue volume fractions between the two groups of patients (Table 2). However, in group B the lamellar bone had a higher mean value than that obtained in group A, where the newly formed bone matrix was mainly represented by less immature matrix, such as osteoid and woven bone.

## 4. Discussion

This research was designed as a randomized controlled trial aiming to answer the following question: what is the suggested treatment for the implant rehabilitation of Elian’s class II residual sockets between non-resorbable, high-density, polytetrafluoroethylene membrane (d-PTFE) left intentionally exposed for 4 weeks after tooth extraction, and socket preservation with anorganic bone material, and extraction followed by guided bone regeneration, performed two months later? The results of the present research failed to find one superior treatment over the other, hence, the null hypothesis of no statistically significant differences between the two tested procedures was accepted.

Although the present research results are similar between groups, the main benefit of the proposed socket preservation technique with a non-resorbable membrane may represent a valid option because it allows for reducing the overall treatment time and costs for the patient. These benefits are mainly due to it may reduce needs for other regenerative techniques, without impairing the final results, the predictability of the implant treatment, and last but not least, patient satisfaction.

Intact buccal plate (Elian class 1 defect) has been considered a prerequisite for soft-tissue stability in the area surrounding the fixture and, therefore, for long-term esthetic results, particularly in case of high aesthetic demands [20,21,22]. Continuous resorption of the thin buccal bone plate may lead to a high risk of gingival resorption in time [5,20]. The treatment of anterior defects often requires a second surgery consisting of additional bone regeneration procedures to allow for a prosthetically guided insertion of the implants. However, these procedures are usually not more acceptable to the patients due to their invasiveness and cost. The proposed technique of using a non-resorbable membrane to repair the damaged buccal wall temporarily, and intentionally left exposed above the grafted socket, seems to be promising and straightforward, requiring fewer surgical procedures. In the present research, no differences were found regarding the histological quantitative examination. In fact, the buccal plate reconstruction with an intentionally exposed non-resorbable membrane seems to be an effective and easy procedure for regenerating a resorbed buccal bone plate, potentially reducing the need for guided bone regeneration. The proposed technique may be strongly accepted by patients due to its reduced invasiveness.

The biological reason to wait four to six weeks before d-PTFE membrane is to wait until the end of the initial healing phase, before the bone remodeling process. At this stage, under the d-PTFE membrane, it is possible to expect a complete soft tissue closure. The d-PTFE membrane may act like a scaffold to guide the new soft tissue healing, preserving the convex shape of the alveolar crest. On the contrary, in the GBR group, a period of eight weeks before completely recovering of the soft tissue is recommended, according to Buser and co-authors [18].

The non-resorbable membrane works as a barrier allowing the separation of the soft tissue from the bone for 4 to 6 weeks, the timing needed to reach the stabilization of the blood clot [23]. The removal of the non-resorbable membrane after this period seems to give sufficient time to allow inside the socket the differentiation of mesenchymal cells into osteoblasts, excluding fibroblasts from the gingival flap, and finally leading to new bone formation and maturation. For this purpose, it is crucial that the patient keeps the surgical wound clean and disinfected. However, similar suggestions were given for both techniques. Compared with staged guided bone regeneration, the presented technique allows delivery of the definitive restoration in a shorter time, potentially improving the acceptance of the patients, and also potentially reducing the overall cost.

In a histological study in humans, a biopsy was taken after 4 weeks at the moment of membrane removal. The results of this histological research demonstrated that, even if the non-resorbable d-PTFE membrane was left intentionally exposed, no epithelial tissue over a dense connective tissue matrix was found [24]. This finding may indicate that the newly formed connective tissue seems to be a well-vascularized osteoid matrix. Nevertheless, it needs more time to complete maturation and become a mineralized tissue, allowing for implant placement [25]. An overall period of 3 to 5 months is necessary. Basically, it depends on the type and size of the defect and the biomaterial used to graft the socket. In another histological study, the test group received a combination of 70% mineralized and 30% of demineralized cortical allograft material, used to graft a post-extraction socket covered with an intentionally exposed, non-resorbable d-PTFE membrane. The obtained results were evaluated site by site with a control group, for which only a mineralized allograft material was used [26]. The biopsies demonstrated an increased vital bone formation (36.16%) with consequential reduction of the residual graft (18.24%) in the test group, compared with the controlled cases, with 100% of mineralized bone allograft group (24.69% and 27.04%, respectively) [19,26,27].

The results of the present study are in agreement with a recent Cochrane systematic review that ended concluding that alveolar ridge preservations techniques may minimize the overall changes in residual ridge height and width after teeth extraction. Nevertheless, evidence is very uncertain [15].

Due to the lack of sample size calculation, the main limitation of the present study was the lower sample. Another limitation, which may alter the internal and external validity of the results, was the lack of surgeon calibration. However, all the surgeons involved in this research were trained in performing two explanatory cases before starting the trial. Finally, maxilla and mandible have different bone resorption rate and pattern, which may affect the prognosis. However, randomization allowed to have two balanced groups, with no statistical differences between them. Therefore, the results of the present preliminary report provide quantitative histological data of bone reconstructed with non-resorbable d-PTFE membrane, or guided bone regeneration for the treatment of post-extractive alveolar bone defects, with implant-supported restorations. Although the proposed open wound healing technique has been compared with classical guided bone reconstruction, these preliminary results encourage this approach. Likewise, there are studies comparing cellular dermal matrix vs. polytetrafluoroethylene (PTFE) membrane, reaching similar promising results [28]. Therefore, further clinical, and radiological studies, with longer follow-up, are needed to validate these promising clinical results.

## 5. Conclusions

With the limitation of the present pilot study, buccal plate reconstruction with an intentionally exposed non-resorbable membrane seems to be an effective and easy procedure for regenerating a resorbed buccal bone plate, reducing the need for guided bone regeneration. Further studies with larger sample size are need to confirm this preliminary result.

## Figures and Tables

**Table 1:** Main patient and implant characteristics between groups.

	Group A (n = 8)	Group B (n = 10)	*p* Value
Age	58.1 ± 13.2	56 ± 11.6	0.735
Female/male	4/4	7/3	0.631
Smockers	0	0	1.0
Maxilla/Mandible	6/8	8/2	1.0
Maxillary central incisor	3/8	4/10	1.0
Maxillary Canine	1/8	1/10	1.0
Maxillary premolars	2/8	3/10	1.0
Mandibular incisors	0/8	1/10	1.0
Mandibular premolars	2/8	1/10	0.559
Mean implant length (mm)	11.5 ± 0.0	11.1 ± 0.7	0.300
Mean implant diameter (mm)	3.8 ± 0.0	3.8 ± 0.0	N/A

**Table 2:** Mean tissue fractions (%) were computed in the histological samples of the two groups of patients.

	Group A (n = 8)	Group B (n = 10)	*p*-Value
Lamellar bone	16.81 ± 9.61	35.16 ± 12.36	0.077
Woven bone	16.03 ± 4.71	10.12 ± 4.68	0.160
Osteoid matrix	20.78 ± 16.45	11.10 ± 4.13	0.375
Biomaterial	19.92 ± 9.13	13.85 ± 8.31	0.409
Medullary spaces	26.46 ± 18.05	29.77 ± 3.67	0.772

## Data Availability

Not applicable.
